# Contribution to foundations from avian biology foundational studies in Japanese quail and domestic chickens demonstrate conserved mechanisms in reproduction throughout the lifespan

**DOI:** 10.3389/fphys.2026.1976423

**Published:** 2026-10-09

**Authors:** Mary Ann Ottinger

**Affiliations:** Department of Biology and Biochemistry, University of Houston, Houston, TX, United States

**Keywords:** aging, conserved mechanisms, embryonic development, Japanese quail (*Coturnix japonica*), neuroendocrine processes, reproductive endocrinology

## Introduction

Poultry science and more broadly avian biology have provided foundational knowledge for developmental biology, genetics, endocrinology, and neurobiology. Since domestication from the jungle fowl, genetic selection has refined characteristics of domestic poultry strains (Smith and Daniel, 1982). Interestingly even in ancient times, caponization was used to reduce aggression and increase meat quality in males. However, an understanding of hormonal modulators of behavior and metabolism occurred much later, with Berthold’s studies (Berthold, 1849; Sawin, 1996), which provided direct evidence for the effects of testicular secretions in domestic chickens (Soma, 2006). Further, these studies parallel observations on the effects of testicular secretions for humans and other mammals (Handelsman, 2022). Most notable is the study by Brown-Séquard (1889) in which at 72 years of age he experienced rejuvenation from injecting himself with an aqueous extract of guinea pig and dog testes. Although a placebo effect, Brown-Sequard’s findings catalyzed interest in the effects of testicular compounds as a fountain of youth for aging. These studies illustrate the conserved mechanisms of the actions of steroid hormones in mammals and birds and highlight the convergent underpinnings of lifetime reproductive function across vertebrate organisms.

Studies in poultry (*Gallus gallus domesticus; Coturnix japonica)* have provided a cornerstone for understanding developmental biology, neural basis of behavior (ex: testosterone effects, operant conditioning in pigeons), reproduction, disease processes, immune function, and establishing novel research models. Advances in poultry genetics allowed optimization of reproduction in egg layers and growth in meat producing birds. Interestingly, selected strains show significant differences in physiology, including metabolic endocrine characteristics, productivity, immunocompetence, and lifespan. The bursa of Fabricius, described by Bruce Glick and colleagues (Glick, 1966) elucidated its critical role in immune function and demonstrated early steroid induced sex differences in chicks (Glick and Sadler, 1961). Beyond the rich literature on immune function, domestic species have provided tools for evaluating the movement of pathogens in wild birds and risk to domestic poultry (Prosser et al., 2016). This article focuses on conserved mechanisms directing endocrine and behavioral components of reproduction over the lifespan and responding to environmental modulators.

### Embryology

Developmental processes in the chick embryo, first documented by Aristotle have provided the basis for understanding early development (Zagris, 2022). Detailed descriptions of developmental stages in the chick embryo became the standard for recognizing conserved mechanisms across classes of organisms (Hamburger and Hamilton, 1951). With development outside the maternal system, the chick embryo provides a versatile model for studies on molecular development, signaling pathways, differentiation of physiological systems, neurobehavioral pathways, and assessment of toxins. More recently, *in vitro* chick embryo shell-less culture systems provide to opportunity to assess efficacy and toxicity of treatments for cancer on specific cells (Nipper et al., 2024). Transgenic embryos add capability to investigate specific physiological processes throughout embryonic development and over the lifespan (Henderson et al., 2025).

### Reproduction over the lifespan

Sexual differentiation in precocial birds occurs during embryonic development, with. gonadal and accessory gland development preceding activation of the hypothalamic-pituitary-gonadal axis and sexual differentiation of behavior in Japanese quail (**Figure 1**). Altricial birds undergo a more extended sexual differentiation process, notably the sexually dimorphic song system (Adkins-Regan et al., 1990). As shown in **Figure 1**, birds dramatically differ in lifespan, with quail regarded as short-lived and long-lived species such as cranes, condors, vultures, eagles, and seabirds. Further, adult peak productivity varies with lifespan, which can be greatly attenuated by environmental stressors. Importantly, aging processes notably reproductive function mirror those in mammals with conserved mechanisms operating during age-related reproductive decline (Ottinger, 2018; Comizzoli and Ottinger, 2021).

**Figure 1:**
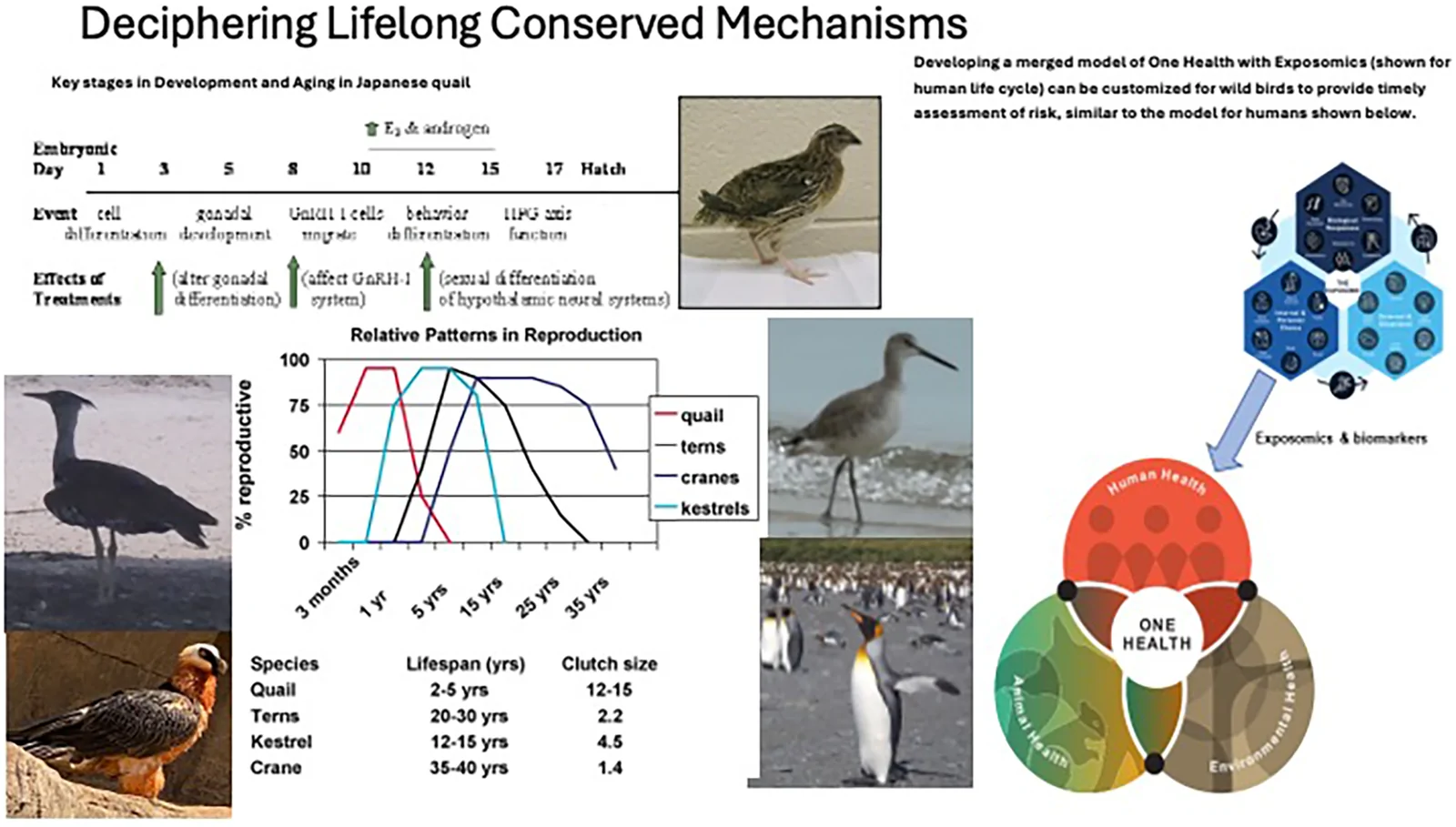
Scientific poster titled “Deciphering Lifelong Conserved Mechanisms” shows bird species images, a lifespan and clutch size table, a reproductive lifespan graph, a key stages in quail development timeline, and diagrams merging One Health and Exposomics models for assessing risk in both birds and humans.

### Environmental factors

Poultry live in a controlled environment, with minimal exposure to environmental chemicals and other stressors experienced by wild birds. In contrast, wild birds experience exposures to mixtures of environmental stressors (pollutants, habitat and resource loss, climate change; Ottinger and Geiselman, 2023). Studies in the domestic chick and Japanese quail have provided a biomarkers of exposure, indicative of adverse physiological and behavioral effects of exposure to contaminants, with precocial species (domestic chicken, Japanese quail, mallard duck) serving as the standard for regulatory testing of industrial, agricultural, and residential use chemicals (Ottinger and Quinn, 2024). Beyond adverse effects of environmental chemicals on reproductive and metabolic systems, toxicants also impair cardiac development, chick locomotor and vocal behaviors, and molecular markers of toxicants (Quinn and Ottinger, 2008; Carro et al., 2013, 2018; Legrand et al., 2021; Ottinger and Dean, 2022). Field birds are impacted by environmental crises, such as the Gulf oil spill (Perez et al., 2017). Using these biomarkers of exposure also provide the basis for developing a field friendly ‘tool kit’ for wild birds (Ottinger and Dean, 2022; Grace et al., 2024; Ottinger and Quinn, 2024). Further, many long-lived species have 1–2 chicks/year so that any adverse effects from pollutants and other stressors can severely impact population resilience (Kruger et al., 2022; Grace et al., 2024).

## Discussion

Avian Biology has provided the platform for understanding embryonic development and neuroendocrine processes modulating sexually dimorphic behavior. Unique metabolic and respiratory system adaptations for flight and migration, bone physiology, and vestibular hair cell regeneration provide valuable models systems. Conserved Mechanisms are the hallmark of contributions of poultry science to vertebrate biology and biomedicine. In combination with insights associated with genetics and embryology, immunology, neural development, reproduction, and aging processes have produced essential information and platforms for understanding molecular mechanisms and biomedical applications.

### One Health conceptual framework

Birds are indicators of ecosystem resilience and overall planetary health and occupy many niches. For example, vultures play a critical role as scavengers (Wink et al., 2026). The concept of One Health encompasses these and other interrelationship of humans, wildlife, and the environment (**Figure 1**). Utilizing bioindicators of exposure (Exposome and exposomics; **Figure 1**) will enhance predicting risk and lifetime health (healthspan; Ottinger et al., 2024; Ottinger and Geiselman, 2025). Building on the foundation of poultry research will ensure the health of domestic species and build an effective platform for conserving wild avian species.
